# Criteria of medical students for the selection of their future clinical specialisation: a cross-sectional survey at the Medical Faculty of Rostock

**DOI:** 10.3205/zma001284

**Published:** 2019-11-15

**Authors:** Anke Gebhard, Brigitte Müller-Hilke

**Affiliations:** 1 University of Medicine of Rostock, Institute for Immunology, Rostock, Germany

**Keywords:** human medicine, education, specialist selection

## Abstract

**Objective:** Despite the increase in the number of graduates in Human Medicine, it has been predicted that there will not only be a shortage of doctors, but also a shortage of specialists in Germany for the years to come. At the same time there are only a few studies on the factors that influence medical students in their decision to select a specialty. Against this background, a study was conducted at the Medical Faculty in Rostock, to investigate the criteria for deciding on a later field of specialty.

**Methods: **Conducting focus groups of four to seven participants of each year of study, criteria were determined which seemed relevant to the students during their selection of their specialty field. The interviews were transcribed and the answers of the participants classified in super categories. With the data obtained a questionnaire was prepared and sent electronically to all students of Human Medicine. Via exploratory factor analysis important criteria and their correlations were determined.

**Results: **A total of 421 students took part in the questionnaire (31.4% return) and indicated their own clinical experience, but also patient contact and work-life-balance as important criteria for a future selection of specialty field. 44.8% of the participants had already made their choice of specialty at the time of the query, and this for the most part took place in the clinical study phase and most often for the subjects Internal Medicine (15.3%), General Medicine (14.2%), Pediatrics (12.0%) and Surgery (11.5%). For those students who already had made a decision, the size and complexity of the field as well as the opportunity to be able to build relationships with patients, were decisive for selection. Those still undecided indicated that for their choice of specialty family friendliness of the field, leisure time and esteem from others were important.

**Conclusions: **Our results show that the personal clinical experience can be a decisive influential factor for future specialty selection. Early contact with the different disciplines could therefore be structured as an aid to help in the decision making, in order to break down any apprehensions and to stop a pending deficiency in specialists.

## Zusammenfassung

**Zielsetzung: **Trotz steigender Absolventenzahlen im Studiengang Humanmedizin wird für die kommenden Jahre nicht nur ein Ärzte-, sondern auch ein Facharztmangel in Deutschland vorhergesagt. Gleichzeitig gibt es nur wenige Studien darüber, welche Faktoren Medizinstudierende bei der Facharztwahl überhaupt beeinflussen. Vor diesem Hintergrund wurde an der Universitätsmedizin Rostock eine Studie durchgeführt, um die Entscheidungskriterien für die spätere Facharztwahl zu ergründen.

**Methoden: **In Fokusgruppen von je vier bis sieben Teilnehmern jeden Studienjahres wurden Kriterien ermittelt, die den Studierenden bei ihrer Facharztwahl als relevant erschienen. Die Interviews wurden transkribiert und die Antworten und Argumente der Teilnehmer in Überkategorien eingeteilt. Anschließend wurde mit den gewonnenen Daten ein Fragebogen erstellt und an alle Studierenden der Humanmedizin elektronisch versandt. Mittels explorativer Faktorenanalyse wurden wichtige Kriterien und ihre Zusammenhänge ermittelt.

**Ergebnisse:** Insgesamt nahmen 421 Studierende an der Umfrage teil (31,4% Rücklauf) und gaben die eigene klinische Erfahrung, aber auch Patientenkontakt und Work-Life-Balance als wichtigste Kriterien für eine zukünftige Facharztwahl an. 44,8% der Teilnehmer hatten zum Zeitpunkt der Befragung ihre Facharztwahl bereits getroffen, wobei dies mehrheitlich im klinischen Studienabschnitt stattfand und am häufigsten für die Fächer Innere Medizin (15,3%), Allgemeinmedizin (14,2%), Pädiatrie (12,0%) und Chirurgie (11,5%). Bei bereits entschiedenen Studierenden waren die Größe und Komplexität des Fachs sowie die Möglichkeit, eine Beziehung zum Patienten aufzubauen, ausschlaggebend für die Wahl. Die noch nicht Entschiedenen gaben an, dass für ihre Facharztwahl die Familienfreundlichkeit des Fachs, Freizeit und die Meinung anderer wichtig sein würden.

**Schlussfolgerungen:** Unsere Ergebnisse zeigen, dass die eigene klinische Erfahrung die zukünftige Facharztwahl maßgeblich beeinflussen kann. Früher Kontakt mit den verschiedenen Fachrichtungen könnte daher strukturiert als Entscheidungshilfe eingesetzt werden, um etwaige Vorurteile abzubauen und drohenden Facharztmangel abzuwenden.

## Introduction

After graduating, medical students have to decide on the specialty field they would like to work in as future doctors. In Germany, you can choose between 33 different fields, and according to current statistics of the German Medical Association, most doctors work in Internal Medicine (53,362=13.9%), followed by General Medicine (43,524=11.3%) and Surgery (36,991=9.6%) [1]. Although the numbers of doctors have continued to increase over the recent years, it is predicted that due to the increase in demand there will be a doctor and specialist deficit in Germany in the years to come. Reasons for this increase in demand of medical care are among others the increase in life expectancy of the population, the reduction of working hours and an increase in job opportunities. Especially in General Medicine the problem becomes clear, since scarcely populated regions are increasingly lacking in resources [2], [3]. 

In order to prevent a deficiency in specialists and to make supposedly undesirable specializations more attractive to young doctors, the criteria which could be the decisive factor for a specialty must be made known. While there are only few German studies on this, international publications of retrospective surveys of doctors indicate that role models with professional conduct and expertise [4] in addition to compatibility between profession and family [5], personal skills, contact with patients and clinical experiences all play important roles [6]. In the present cross-sectional study, students at the University Medicine in Rostock were asked about their current mood with regard to their future choice of specialty. Our goal was to find out what criteria the students named in this early stage of decision making. The following questions were relevant:

- Which criteria influence the selection of a specialty?
- What fields of specialty are most popular among students?

## Methods

### Interviews

Data collection and processing was performed according to the “Grounded Theory” [7]. In a first approach, we conducted partially standardized interviews in focus groups of four to seven participants each. Participants were recruited from the second to sixth year of studies by distributing self-designed fliers, by word of mouth and by directly approaching the students after classes. The key questions of the interviews were:

- “Have you decided on your specialty already?”
- “If yes, for which specialty have you decided on and how did you decide for this?”
- “If no, why haven’t you decided yet?”

All interviews were then transcribed verbatim, coded using the MAXQDA software (Verbi software GmbH, Berlin), and the criteria denoted were classified in super categories. The local ethics committee declarer out study unobjectionable (A 2016-0186).

#### Questionnaire creation

Using the collected criteria and their super categories, a questionnaire was created with the Evasys program, which asked study participants about their demographic data and included 36 questions on the criteria for specialist selection. The question types were open as well as closed. For closed questions a dichotomous answer scheme was stipulated, but also answers on a scale of 1-5 (1: not important/ does not apply, 5: very important/ relevant). The questionnaire was sent in the summer semester of 2017 by the dean’s office of Rostock as an online survey to all students of the University Medicine Rostock. The questionnaire can be found in the attachment 1 .

#### Statistical Analysis

The survey was analyzed by Microsoft Excel (Microsoft Corporation, Redmond, WA, USA) and IBM SPSS (Armonk, NY, USA). The Gaussian distribution of the data was tested using the Kolmogorow-Smirnow Test. In order to be able to make a statement about the reliability, Cronbach’s alpha was calculated. X^2^-Tests (questions with dichotomous answer options) and Mann-Whitney-U-Tests (questions with scaled answer options) were conducted in order to compare students who have already made a decision and those who were still undecided. In order to check the compatibility of the dataset for factor analyses, the Kaiser-Meyer-Olkin-Measure and the commonalities were determined and the anti-image covariance matrix was analyzed. A total of 47 variables was taken into consideration. An exploratory factor analysis (main axis method) was conducted with Varimax rotation in order to interpret and classify the criteria. To determine the number of factors, the Kaiser-Guttmann criterion and the Scree Test were applied.

## Results

### Development of the questionnaire and description of the study collective

With the goal to identify as many criteria for the specialist selection as possible, five partially-structured focus group interviews were conducted with a total of 25 representatives of study years two through six each, whereby 21 of the representatives were female. The analysis of this interview confirms a saturation of the arguments and permits a classification of the relevant criteria for the selection of a specialty in the following six super categories:

1. personal skills/ personal character,
2. personal experiences,
3. Work-Life-Balance,
4. role models,
5. professional perspectives and
6. expectations/esteem from outside.

From these super categories a questionnaire was created with a total of 36 questions which were then sent electronically to all students of Human Medicine at the University Medicine. Calculating the internal consistency of the questionnaire resulted in a value of 0.61 for Cronbach’s alpha.

A total of 421 students took part in the survey. With an average year size of 224 students the mean response was 31.4% with slight increases in the 4^th^ and 5^th^ year of study amounting to 36.6 and 40.6%, respectively. The average age of the participants was 23.7±3.4 years and more than 70% were female. 

#### Results of the exploratory factor analysis

In order to contemplate the different arguments in relation to each other and to highlight the most important ones, an exploratory factor analysis was conducted. The results of this analysis are summarized in table 1 (Tab. 1) and show that eight components comprise the majority of the total variance and therefore were relevant criteria for the specialty selection. The table sorted these eight components according to their portion of eigenvalue to the total variance and lists the contained criteria according to decreasing rotated factor loading. At the same time only such criteria were listed, whose rotated factor loading was greater than 0.5. With an eigenvalue of 8.3% the most relevant criterion for participants of our survey was their personal clinical experience when deciding for or against a certain field. These components were influenced by the semester as well as the time of specialty selection and included clinical traineeships, clinical internships and contact with doctors. Additional relevant components were contact with patients, work-life-balance, rational aspects such as income and job security, esteem from outside, influence of the teaching, the size of the respective field as well as gender. These 8 components comprised 39.1% of the overall relevance. 

#### Time of decision

**Figure 1:**
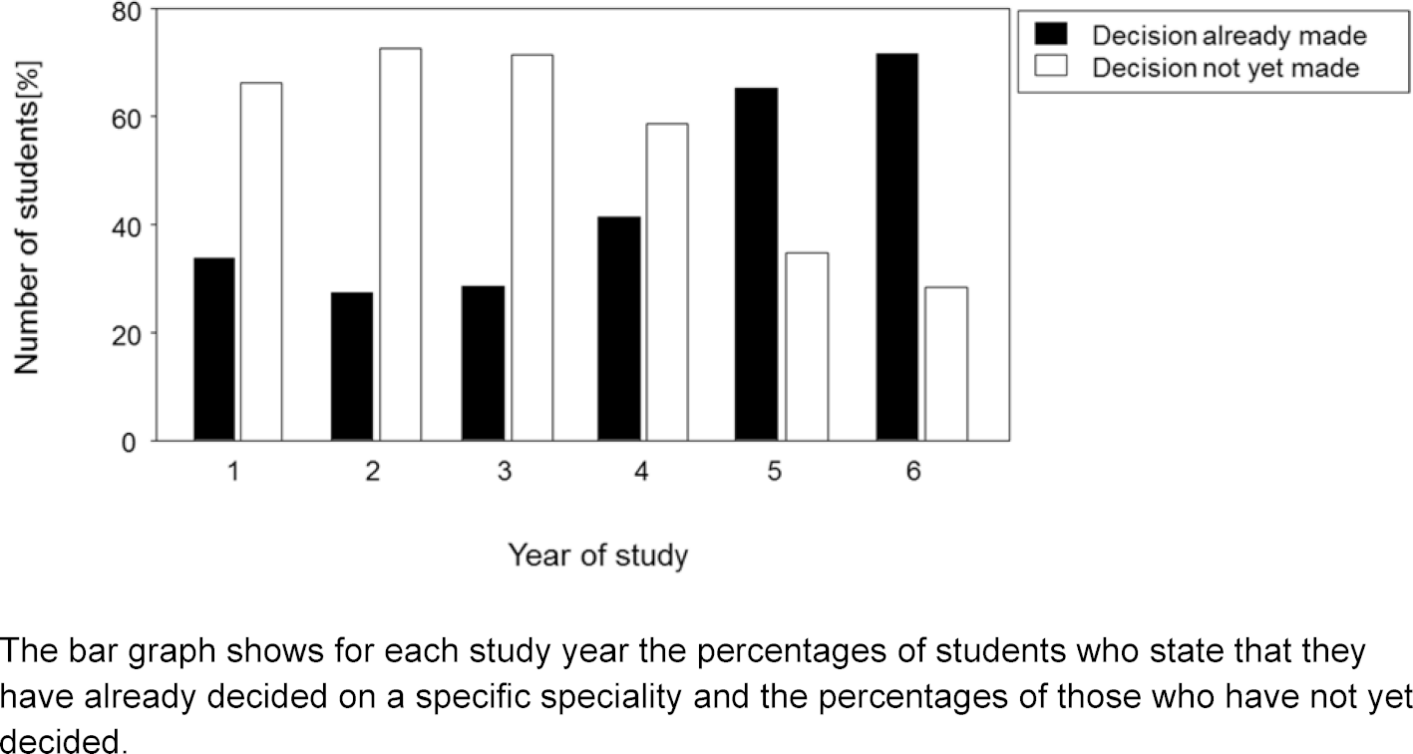
The bar graph shows for each study year the percentages of students who state that they have already decided on a specific speciality and the percentages of those who have not yet decided.

At the time of the survey 44.8 % of all participants had already decided on their specialty. In figure 1 (Fig. 1) it can be seen that approximately one third of those beginning their studies came to the university with a clear vision and were more set on a specific specialty than the second and third-year students. The decision to become a specialist increased later on in the course of the clinical semesters and in the fifth year of study, the students who had already made a decision outweighed the ones who had not. 

#### Desired fields of specialty 

**Figure 2:**
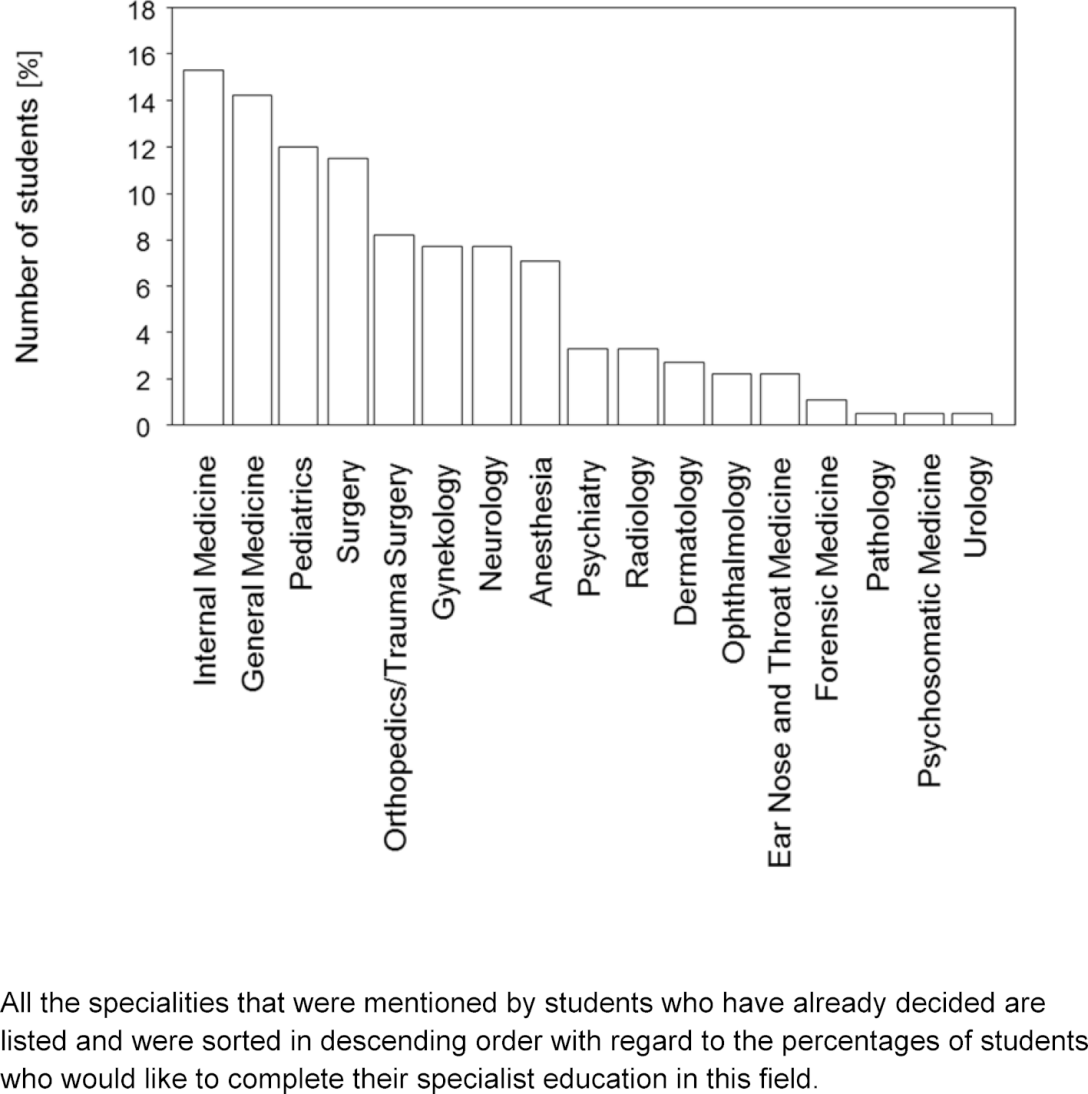
All the specialities that were mentioned by students who have already decided are listed and were sorted in descending order with regard to the percentages of students who would like to complete their specialist education in this field.

From the 188 participants who had already decided on a field, 183 disclosed the field they were pursuing. All subjects mentioned are summarized in figure 2 (Fig. 2). The most common subjects mentioned were Internal Medicine (15.3%), General Medicine (14.2%), Pediatrics (12.0%) and Surgery (11.5%). However, there was a significant difference among the genders. While the female participants predominantly chose fields like Pediatrics, General Medicine and Gynecology, male students most often chose Internal Medicine, Orthopedics/Trauma Surgery and General Medicine as their preferred field.

**Figure 3:**
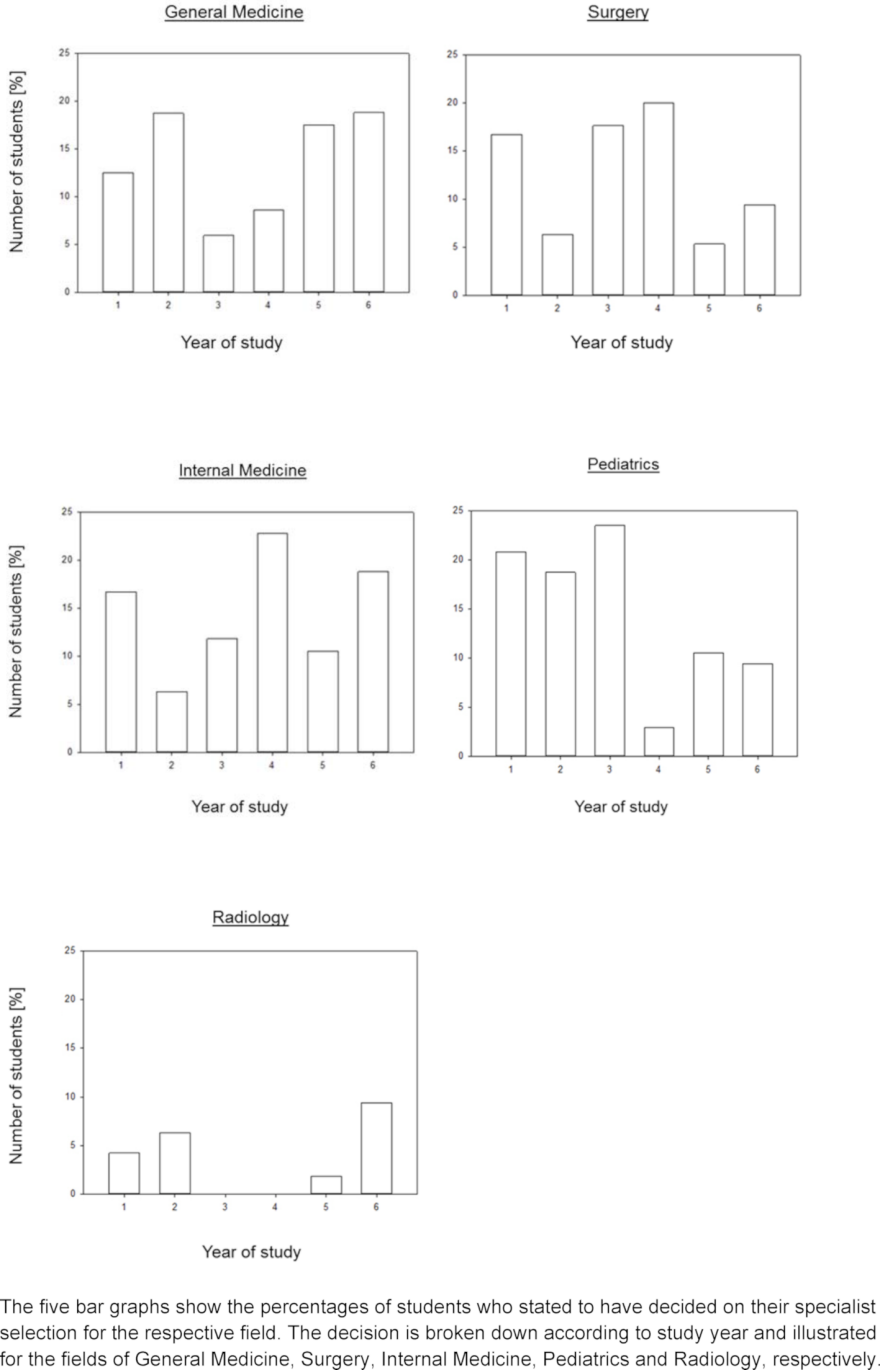
The five bar graphs show the percentages of students who stated to have decided on their specialist selection for the respective field. The decision is broken down according to study year and illustrated for the fields of General Medicine, Surgery, Internal Medicine, Pediatrics and Radiology, respectively.

In figure 3 (Fig. 3) the decision for a field is displayed in correlation with the respective year of study using five specialties as an example. In each of the five subjects there are significant fluctuations visible in the course of the studies. But also between the individual fields differences can be seen. For example, the enthusiasm for Pediatrics is especially great in the first three years of study and then decreases, whereby in General Medicine the enthusiasm increases steadily from the third year of study. Surgery is most popular in the third and fourth study years, Internal Medicine is also popular in the fourth year of study, whereas interest for Radiology increases only at the end of the studies. 

#### Comparisons between students who have already decided and those still undecided 

In order to examine whether different criteria play a role in specialty selection for those students who have already decided and those still undecided, X^2^-Tests were conducted for questions with dichotomous answer options and Mann-Whitney U Tests for questions with scaled answer options. The results of these two tests are presented in table 2 (Tab. 2) and table 3 (Tab. 3) and examine the same questions, only that due to the different question types, different tests had to be applied. Both tables present significant differences between those students who have already decided and those still undecided with regard to the criteria relevant for them for specialty selection. Table 2 (Tab. 2) shows that for different students the size and complexity of the field for the specialty selection was decisive, whereby for those still undecided aspects like family friendliness and leisure time were paramount for a later choice. Table 3 (Tab. 3) shows further that for those undecided, the esteem of others as well as TV series were significantly more relevant criteria for their later specialty selection as for those who had already made their decision for a specific field. In contrast those who had already decided on their field, the opportunity to build patient relationships was a significant relevant decision criterion. For the sake of completeness table 3 (Tab. 3) lists the criteria “family influences” and “positive feedback” however, the p value indicates subordinate roles. It was also to be seen that students who were still undecided considered more criteria as important on the whole as those who had already decided on their specialty.

## Discussion

Our study shows that for the students their personal clinical experience played an important role for their future specialty selection. Decisive were clinical traineeships, clinical internships and contact to doctors. Decisive was also whether the respective specialty allowed the building of a relationship to the patient, direct patient contact or talks with patients. Important for the specialty selected during the course of studies was also the Work-Life-Balance. In addition we could show gender-specific differences and previous studies confirm that Internal Medicine, General Medicine, Pediatrics and Surgery turned out to be the most popular fields [8], [9]. Unexpected was the result that for those who had already decided on their choice of specialty and for those who were still undecided, completely different criteria were relevant. Whereas with those who had already decided the size and complexity of the respective field as well as the ability to build relationships to patients were guiding factors and a certain amount of enthusiasm, while those still undecided struggled with the Work-Life Balance and the esteem of others. Questionable is whether both of these aspects actually represent relevant reasons for those students still undecided or whether they just haven’t had the chance to become enthusiastic about a certain field. Our students also indicated for example that the personal clinical experience was decisive for their current decision for or against a certain specialty field, where structured decision aids can be applied in the future. In the regular study course clinical experience is gained in self-selected clinical traineeships or in the internships. However our courses of enthusiasm for the different fields (see figure 3 (Fig. 3)) show that the compulsory lessons can have very different effects. The enthusiasm for Surgery at the time of the lecture (3^rd^ and 4^th^ year of study) is significantly higher than at the time of the block internship (5^th^ year of study) or in the practical year. The enthusiasm also decreases for the field of Pediatrics with the onset of the lecture in the 4th year of study and the following block internship. 

From the USA there are already examples for programs for career planning, such as for example the “Selectives” of the Mayo Clinic. Goal of these one to two-week block events is to help med students through workshops, podium discussions and clinical insights in their decision for a later specialization [10], [11]. Comparable is also the offer of the Columbia University which already offers from the first semester informal talks, observations in several fields, individual career consultation interviews and discussion rounds [12]. Also the University of Michigan Medical School offers support in the area of career planning, which focus on self-evaluation exercises under the guidance of professional consultants and mentoring by students of higher semesters, which lead to a significantly higher degree of satisfaction in all areas of career planning in students [13]. It is plausible to establish such programs in German universities as well.

For Germany a structured support for career planning has only been described on an exemplary basis up to now [14]. For example, the Medical Faculty of the Ludwig-Maximilians-University Munich (LMU) offers a mentoring program that could be further developed and tested throughout the country [15]. Another possibility to grant insight into the different fields and to eliminate possible prejudice at the same time is offered by so called “specialist duels” [16]. Since students who are still undecided take more criteria into consideration when selecting their specialty than those who have already decided, such information events would be helpful in order to eliminate any prejudice or misconceptions. With our study participants the specialist selection was made mainly in the fifth and sixth year of study, whereby this relatively late point in time could be the result of our regular curriculum. It would be tempting to speculate if the specialist selection is made earlier in model curricula with an early clinical reference and if similar criteria play a role. As a general rule it makes sense to consult students early enough and to give them the opportunity to gain practical experience [17]. Especially fields in which a lack of doctors can be predicted or is already evident, practical experience could be promoted earlier in the course of studies which could result in more students wanting to go into the field. As a result, the master plan of 2020 intends to restructure the medical studies and to design it with a more practical character. The doctor-patient communication and the doctor-patient relationship will be given more attention and subjects like General Medicine will be strengthened [18].

In the years to come it will be seen whether this can counter the lack of doctors or if additional action must be taken. 

A limitation of this study exists in that we asked with our interviews and in questionnaires about criteria for the specialist selection, however, we do not know whether the criteria collected with the exploratory factor analysis only represent a transient mood in the course of the studies. How stable these supposedly made decisions are and to what extent these decision criteria mentioned actually influence the later specialty selection, will be examined more closely in a long-term study on an immatriculated year of the University Medicine Rostock. For example one could also find out how often and why students change their minds during their studies. Since we have only conducted our study on one faculty, we cannot currently generalize our data. However a cross-sectional study of all practicing doctors from Finland shows that the decision criteria mentioned by our students such as for example experiences during the studies, size and complexity of the field, family friendliness, social standing, as well as demand and job security are also internationally relevant [19].

A decision whether and what consequences result from the data collected at the University Medicine Rostock, has not been made. It is also plausible to further develop existing programs such as the loyalty scholarship in Mecklenburg-Vorpommern for aspiring general practitioners or “specialist duels”, in order to offer students decision aids during their studies.

## Acknowledgement

We thank all students of the University Rostock who took part in the interviews and the survey.

## Competing interests

The authors declare that they have no competing interests. 

## Supplementary Material

Sample: questionnaire for speciality selection

## Figures and Tables

**Table 1:**
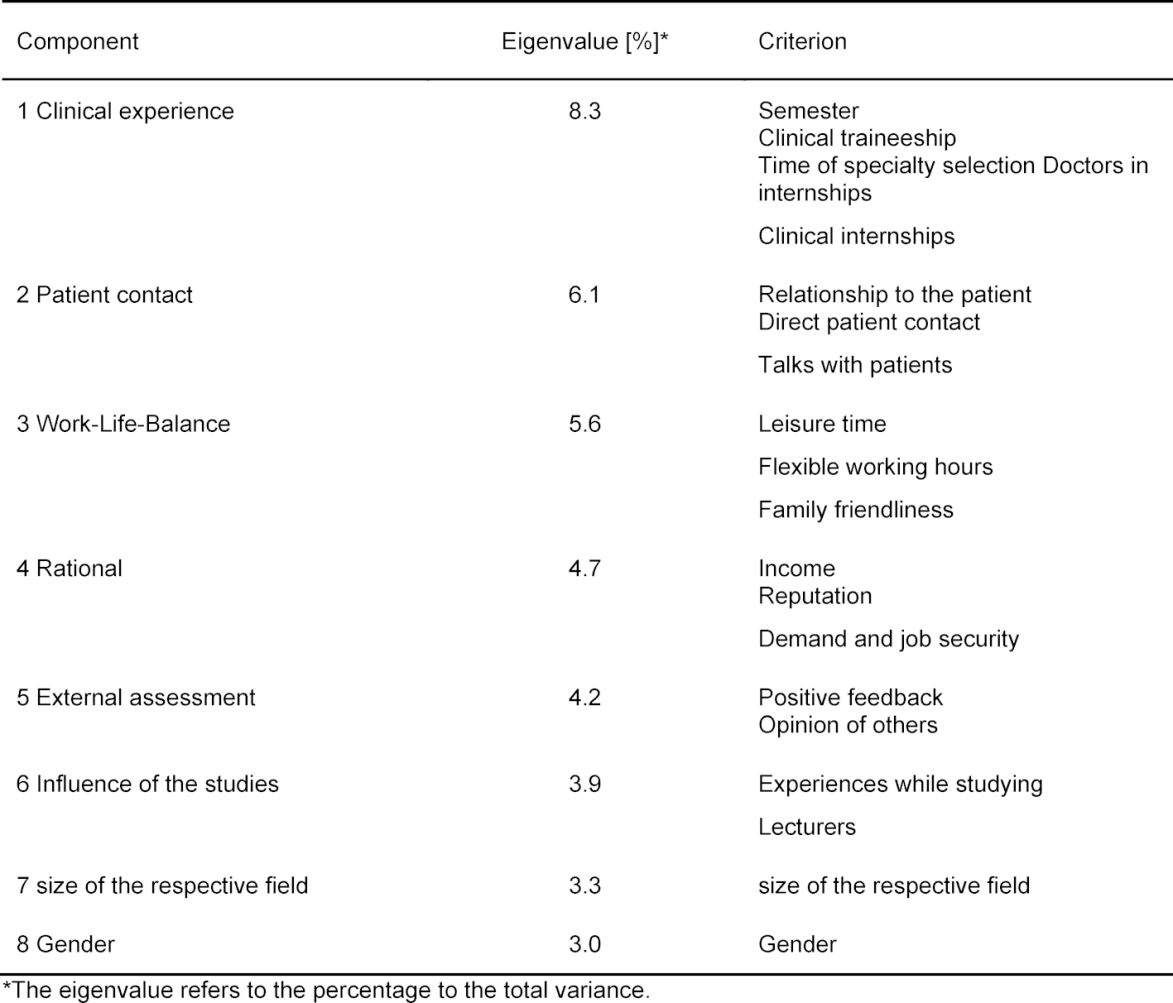
Results of the exploratory factor analysis.

**Table 2:**
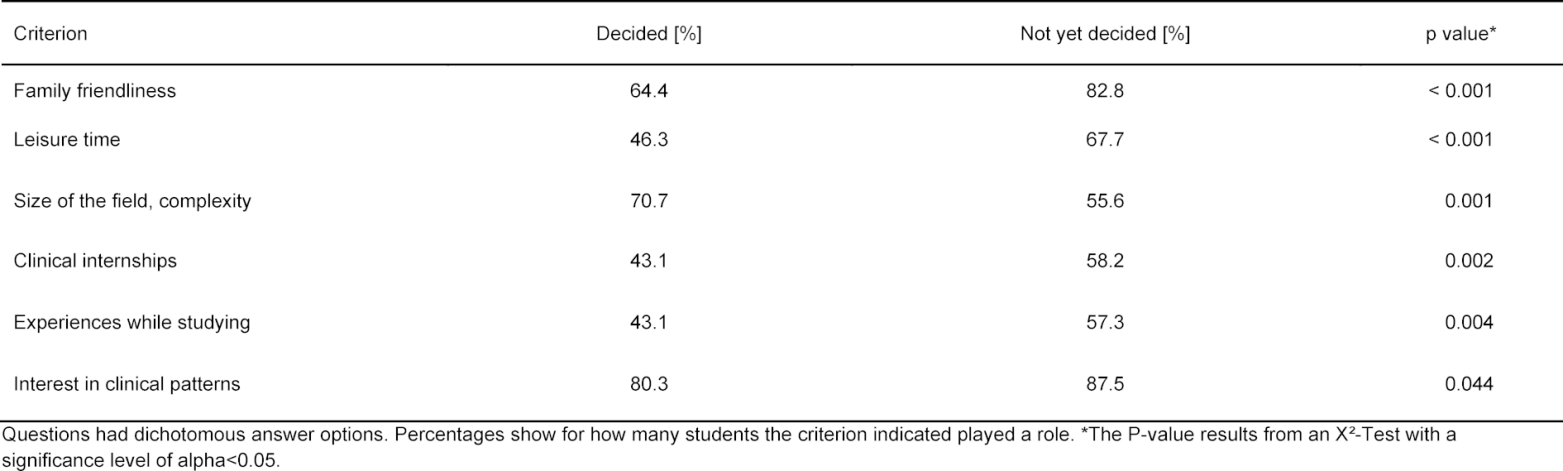
Criteria for the specialty selection (questions with dichotomous answer options).

**Table 3:**
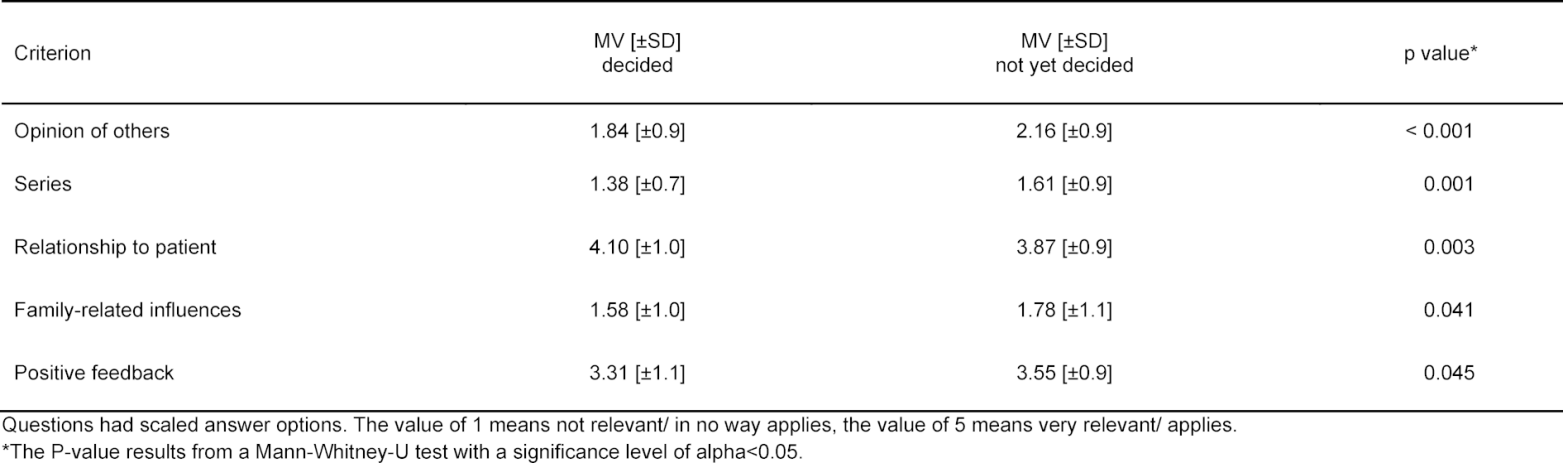
Criteria for the specialty selection (questions with scaled answer options).
