# Supplementary material for: Criteria of medical students for the selection of their future clinical specialisation: a cross-sectional survey at the Medical Faculty of Rostock
Source: GMS J Med Educ. 2019 Nov 15;36(6):Doc76. doi: 10.3205/zma001284 (PMC6905363; doi:10.3205/zma001284)
Supplement: Sample: questionnaire for speciality selection [file JME-36-6-76-s-001.pdf]

SAMPLE

EvaSys	Questionnaire for speciality selection	Electric Paper

Institute for Immunology — Anke Gebhard
WG Teaching Research — Questionnaire for speciality selection - What factors

Please mark as such: ☐ ☒ ☐ ☐ ☐ Please use a ballpoint pen or a felt tip pen that is not too thick. This questionnaire will be read by machine.
Correction: ☐ ■ ☐ ☒ ☐ In the interest of optimum data collection please note the information provided on the left when filling out.

1. General data

1.1 Age:

1.2 Gender: ☐ female ☐ male

1.3 Subject semester ☐ 4. ☐ 6. ☐ 8. ☐ 10. ☐ 12.

1.4 Over which quote were you admitted into the medical study program? ☐ Sum. Cum Laude ☐ Waiting period ☐ Selection method of the university with a selection interview ☐ Selection method of the university without a selection interview ☐ other

1.5 Have you already decided on a field of study? ☐ Yes ☐ No

1.6 In what field would you like to work later? ☐ curative ☐ non-curative

1.7 In which field of study would you like to work as a physician after your studies?

1.8 When did you make that decision? ☐ already before my medical studies ☐ during the pre-clinical phase ☐ during the clinical phase ☐ during my practical year

2. Own skills/ own character

2.1 What is important to you in your future field? (multiple answers possible)
☐ manual/operative work
☐ frequent handling of medication and finding an optimum dosage for the patient
☐ thinking/contemplating/ solving difficult cases
☐ To be able to have talks with patients
☐ to work with my patients also on a psychological level
☐ other

2.2 ... namely:

SAMPLE

SAMPLE

2. Own skills/ own character [continued]

2.3 What role does direct contact to body fluids (e.g. blood) play for your later professional workday?

I find it great, it is very important to me ☐ ☐ ☐ ☐ ☐ I find it disgusting, do not want to in any case

2.4 Which criteria play an important role for your selection? (multiple answers possible)

- ☐ patient/client base of the field (e.g. only women/ children/ deceased...)
- ☐ direct patient contact
- ☐ the size and complexity of the field
- ☐ interest in the clinical pictures in the field
- ☐ an equal interest in a different field of study
- ☐ the similarity to private interests

2.5 ... namely the following field of study:

2.6 What role does it play for you to select a field in which you can build a relationship with patients?

very important ☐ ☐ ☐ ☐ ☐ not important at all

2.7 How strong does the speed with which you see success in the field influence your choice?

very strong ☐ ☐ ☐ ☐ ☐ not at all

2.8 Does the degree of physical demand play a role for you in the field?

large role ☐ ☐ ☐ ☐ ☐ not at all

3. Own experiences

3.1 How important is fun in your field?

very important ☐ ☐ ☐ ☐ ☐ not important at all

3.2 Which experiences have an influence on your preferences? (multiple answers possible)

- ☐ experiences prior to your studies
- ☐ personal experience with doctors
- ☐ personal diseases / family, friends´ diseases
- ☐ experiences while studying (classes, seminars)
- ☐ clinical internship (block internship, practical year)
- ☐ positive experiences with clinical traineeships
- ☐ pre-clinical internships (e.g. occupational field exploration)
- ☐ other

3.3 ... namely:

4. Work-Life-Balance

4.1 Which role do flexible working hours play in the specialist choice?

very important ☐ ☐ ☐ ☐ ☐ not important at all

4.2 How important is your later income?

very important ☐ ☐ ☐ ☐ ☐ not important at all

SAMPLE

SAMPLE

4. **Work-Life-Balance** [Continued]

4.3 Which circumstances also have an influence on your selection? (multiple answers possible)

- ☐ family friendliness of the field
- ☐ duration and difficulty of the specialist education
- ☐ the amount of leisure time which the field offers outside of working hours
- ☐ other

4.4 ... namely:

5. Role models

5.1 How important for you are/were the people who showed a love for their field? very important ☐ ☐ ☐ ☐ ☐ not important at all

5.2 Who are/were these people? (multiple answers possible)

- ☐ Teachers
- ☐ Doctors in clinical traineeships/ internships/ Practical Year
- ☐ other clinical staff
- ☐ parents/Family
- ☐ other

5.3 ... namely:

5.4 To what extend do TV series influence you when making your decision? very much ☐ ☐ ☐ ☐ ☐ not at all

5.5 What series are/were those?

6. Professional perspectives

6.1 Make an X where applicable. (multiple answers possible) important for me is/are:

- ☐ existence of the specialty in my choice location
- ☐ the jobs offered
- ☐ future need in the specialist field/ job security
- ☐ the opportunity to open a practice
- ☐ other

6.2 ... namely:

7. Expectations / estimations from outside

SAMPLE

SAMPLE

7. Expectations / estimations from outside [continued]

7.1	How strongly were you influenced by others whether you have received positive feedback in a field?	very much	☐	☐	☐	☐	☐ not at all
7.2	To what extent do family influences affect you (e.g. possible takeover of a practice/ pressure from family)?	very much	☐	☐	☐	☐	☐ not at all
7.3	What role does the opinion of others play with regard to your specialist selection?	very important	☐	☐	☐	☐	☐ had no influence on my selection
7.4	How important is your social status?	very important	☐	☐	☐	☐	☐ not important at all

8. A last statement

8.1 Would you like to make one statement with regard to your specialist selection? ☐ Yes ☐ No

8.2 ... namely:

SAMPLE
